# Structure and electrical properties of BCZT ceramics derived from microwave-assisted sol–gel-hydrothermal synthesized powders

**DOI:** 10.1038/s41598-020-73784-9

**Published:** 2020-11-23

**Authors:** Xiang Ji, Chuanbin Wang, Takashi Harumoto, Song Zhang, Rong Tu, Qiang Shen, Ji Shi

**Affiliations:** 1 School of Materials Science and Physics, China University of Mining and Technology, Xuzhou, 221116 China; 2 State Key Lab of Advanced Technology for Materials Synthesis and Processing, Wuhan University of Technology, Wuhan, 430070 China; 3 School of Materials and Chemical Technology, Tokyo Institute of Technology, 2-12-1, O-Okayama, Meguro-ku, Tokyo 152-8552 Japan

**Keywords:** Materials science, Techniques and instrumentation

## Abstract

A novel microwave-assisted sol–gel-hydrothermal method was employed to rapidly synthesize Ba_0.85_Ca_0.15_Zr_0.1_Ti_0.9_O_3_ (BCZT) powders. The effects of reaction time on the structure, crystallinity, purity and morphology of the products were investigated. The results of XRD, FTIR, SEM and TEM indicated that BCZT powders could be obtained in even 60 min at a low synthesis temperature of 180 °C, which were well-crystallized with stoichiometric composition and uniform grain size (~ 85 nm). BCZT ceramic derived from the rapidly-synthesized powders had a dense microstructure and good electrical properties (ε_m_ = 9579, d_33_ = 496 pC/N, 2P_r_ = 25.22 µC/cm^2^, 2E_c_ = 7.52 kV/cm). The significant electrical properties were closely related to the high activity of the BCZT powders, resulting from the rapid microwave-assisted sol–gel-hydrothermal process.

## Introduction

In the past decades, lead-based materials have been widely used in piezoelectric or ferroelectric fields due to the significant electrical performance. Unfortunately, their applications are being limited worldwide because of the toxicity of lead as well as the related health and environmental issues^1^. As a result, a series of lead-free piezoelectric materials have been developed and paid more and more attention. Among them, Ba_0.85_Ca_0.15_Zr_0.1_Ti_0.9_O_3_ (hereinafter referred to as BCZT) is found to be a lead-free piezoelectric material with excellent electrical properties^2^, which is even comparable to the lead-based materials including the most popular PZT^3^.

As a complex system, the performance of BCZT is dependent largely on the purity, stoichiometry and grain size of the BCZT powders synthesized by different methods. Also, for the preparation of BCZT ceramics, calcination and sintering both require high temperatures. Although most literature reports the preparation of BCZT powder the by solid-state reaction method, relatively higher calcination temperature (> 1300 °C) is necessary^4,5^. Thus, liquid-phase powder precursor synthesis methods deserve study in order to decrease calcination temperature. Even though several liquid-phase synthesis techniques such as sol–gel process, oxalate precursor route and citrate method have been tried to solve the above problems, the issues like high calcination temperature (> 800 °C) still exist^6–9^. Besides, hydrothermal reaction is used to prepare BCZT powders, and the synthesis temperature can be decreased to below 200 °C. Nevertheless, purity issues still limit the popularization of the method, for impurities induced from the complex hydrothermal reaction^10,11^.

Recently, a sol–gel-hydrothermal technique has been reported for powder preparation^12–14^, which combines both the advantages of sol–gel and hydrothermal methods so that the powders with high purity and homogeneity could be obtained at a rather low temperature (< 200 °C). Nevertheless, long reaction time (> 12 h) in the sol–gel-hydrothermal process is still needed^13^.

Microwave-assisted sol–gel-hydrothermal method (hereinafter referred to as MSGH) is a novel technique that can synthesize powders rapidly at low temperature. Based on the sol–gel-hydrothermal process which has been used to synthesis homogeneous and highly pure nano-powders at low temperature^15^, the introduction of microwave may enhance the sol–gel-hydrothermal reaction efficiency and shorten the synthesis time greatly, so that BCZT powders with high purity, uniform grain size and high activity could be well produced^16^. Unfortunately, at the best of our knowledge, rapid synthesizing BCZT powders at low temperatures by MSGH was never reported.

In the present study, a novel technique, MSGH, was employed to rapidly synthesize crystalline BCZT powders with high purity and activity rapidly (60 min) at low temperature (180 °C) for the first time. The effects of reaction time on the structure, crystallinity, purity and morphology of BCZT powders were studied. Furthermore, followed by traditional sintering method, electrical properties of MSGH derived BCZT ceramics were measured, so as to verify the high activity of the powders.

## Experimental

Ba_0.85_Ca_0.15_Zr_0.1_Ti_0.9_O_3_ (BCZT) powders were synthesized by the microwave-assisted sol–gel-hydrothermal method. Firstly, BCZT gel was formed through sol–gel process by using barium acetate (BaC_4_H_6_O_4_), calcium acetate (CaC_4_H_6_O_4_), titanium butoxide (TiC_16_H_36_O_4_) and zirconium butoxide solution (ZrC_16_H_36_O_4_) as the raw materials. The acetic acid solution was mixed with the raw materials and kept stirring at 60 °C for 30 min. The solution turned into sol and then transformed to gel. After dried overnight and grounded, the formed gel was introduced to a NaOH aqueous solution with a concentration of 4 M. The precursor solutions were then sealed and placed in the microwave hydrothermal equipment (JUPITER BF, SINEO, China). The reaction was carried out under 300 W microwave and a synthesis temperature of 180 °C. The precipitate was centrifuged and washed with distilled water and absolute ethanol for several times to remove the soluble impurities. After drying, the BCZT powders were obtained. Finally, the MSGH-derived BCZT powders were pressed into disks, and further sintered at 1400 °C for 2 h to produce BCZT ceramics by traditional sintering method.

The phase structure of the samples was identified by XRD (Rigaku, Ultima III) with Cu-K_α_ radiation, FTIR (Thermo, Nicolet-6700) with the KBr in the range from 400 to 4000 cm^−1^ and Raman spectra (Renishaw, INVIA), respectively. The microstructure was characterized by SEM (Quanta, FEG250) and TEM (JEOL, JEM-2100F). The piezoelectric and dielectric properties were measured by quasi-static d_33_ meter (Institute of Acoustics, ZJ-3AN) and precision LCR meter (Agilent, E4980A). The ferroelectric property was obtained by a ferroelectric test system using a precision LC unit (Radiant, Premier II) at room temperature with a frequency of 10 Hz.

## Results and discussion

**Figure 1:**
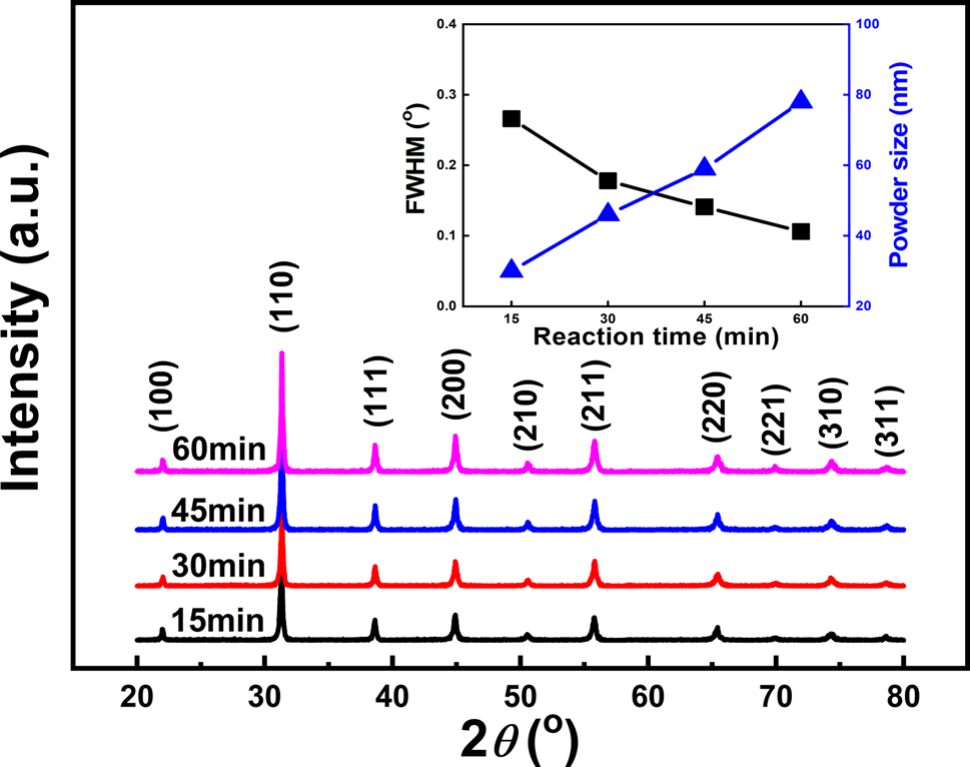
XRD patterns of BCZT powders synthesized at 180 °C for various reaction time [The inset is the FWHM and powder size a function of reaction time].

Figure 1 shows the XRD patterns of BCZT powders synthesized at 180 °C for various reaction time of 15 min, 30 min, 45 min and 60 min. It can be seen that all the powders exhibit perovskite structure with no impurities, suggesting a solid solution formed by doping Ca and Zr into BaTiO_3_ lattice^17^. The inset of Fig. 1 shows the FWHM and powder size of the BCZT powders as a function of reaction time, calculated from the XRD data. Clearly, a better crystallinity could be observed with increasing the reaction time, since the value of FWMH decreases gradually. Also, the powder size calculated by Scherrer formula is estimated to be 29 nm, 46 nm, 59 nm and 78 nm, respectively.

**Figure 2:**
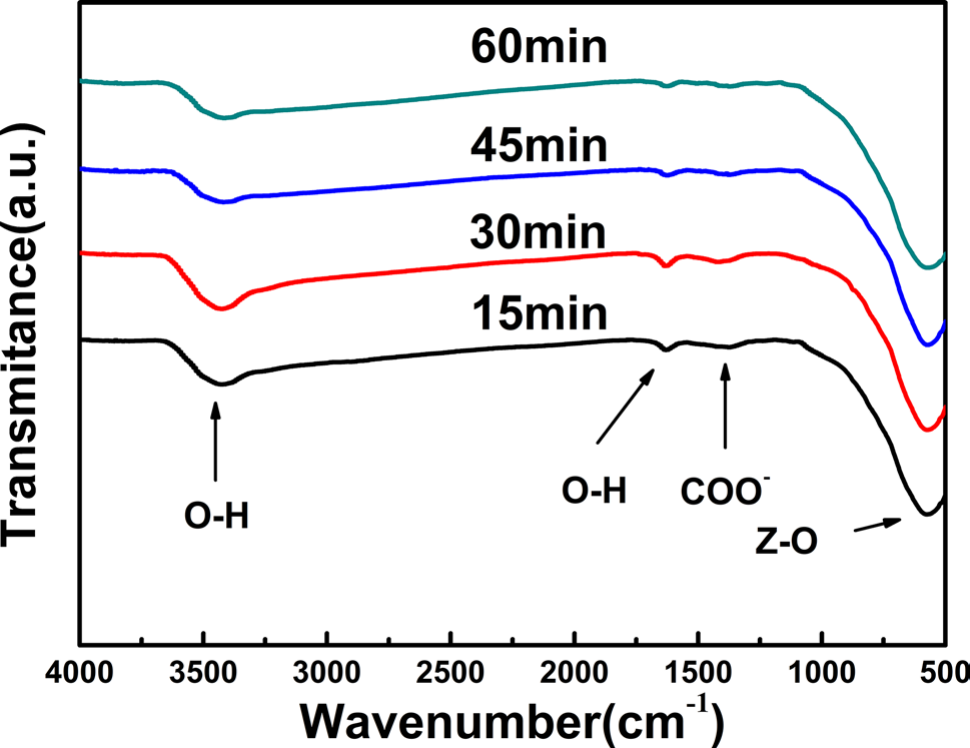
FTIR spectra of BCZT powders synthesized at 180 °C for various reaction time.

Figure 2 shows the FTIR spectra of the BCZT powders synthesized at 180 °C for various reaction time of 15 min, 30 min, 45 min and 60 min. No obvious impurity peaks corresponding to CO_3_^2−^ (around 690, 860 and 1750 cm^−1^) or X–O–C (X = Ti, Zr) groups (around 1120 cm^−1^) can be noted with the extending reaction time^18–20^. Meanwhile, for all the samples, the absorption peaks corresponding to O–H (around 1640 cm^−1^ and 3000–3600 cm^−1^) as well as –COOH (around 1420 cm^−1^) disappear nearly and the peaks corresponding to Z–O (Z = Ba, Ca, Ti, Zr) were quite significant^13^, implying the high purity and crystallinity of MSGH derived BCZT powders^12^.

**Figure 3:**
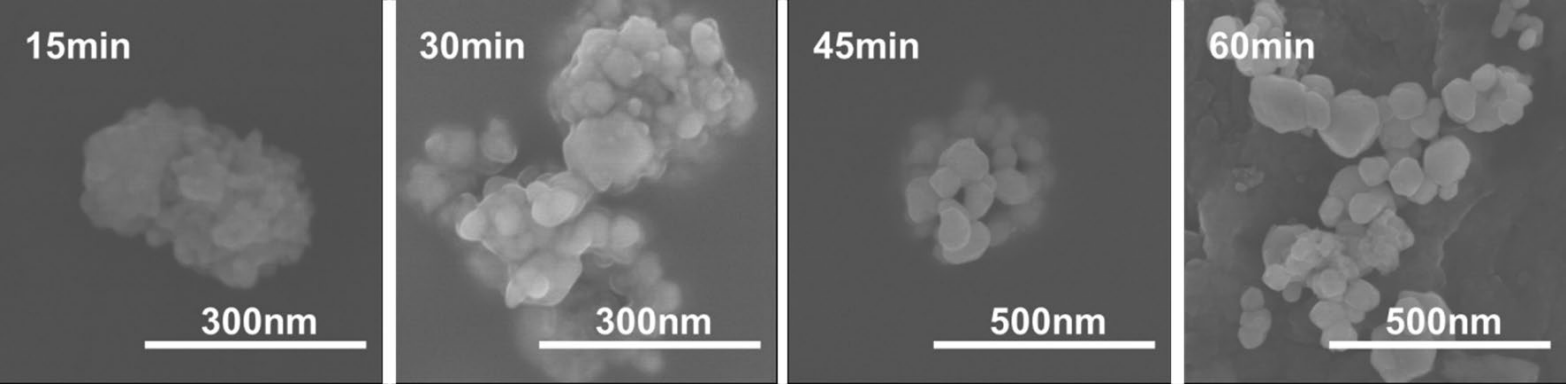
SEM images of BCZT powders synthesized at 180 °C for various reaction time.

Figure 3 shows the SEM images of BCZT powders synthesized at 180 °C for various reaction time of 15 min, 30 min, 45 min and 60 min. For 15 min and 30 min samples, blurred boundaries can be observed with the powder sizes of about 20 nm and 50 nm respectively. With the extending reaction time to 45 min, the powder size increases to about 65 nm. When the reaction time extends to 60 min, the agglomeration is improved and the powder size increases to about 85 nm. The powder size of SEM is close to the calculated value from XRD.

**Figure 4:**
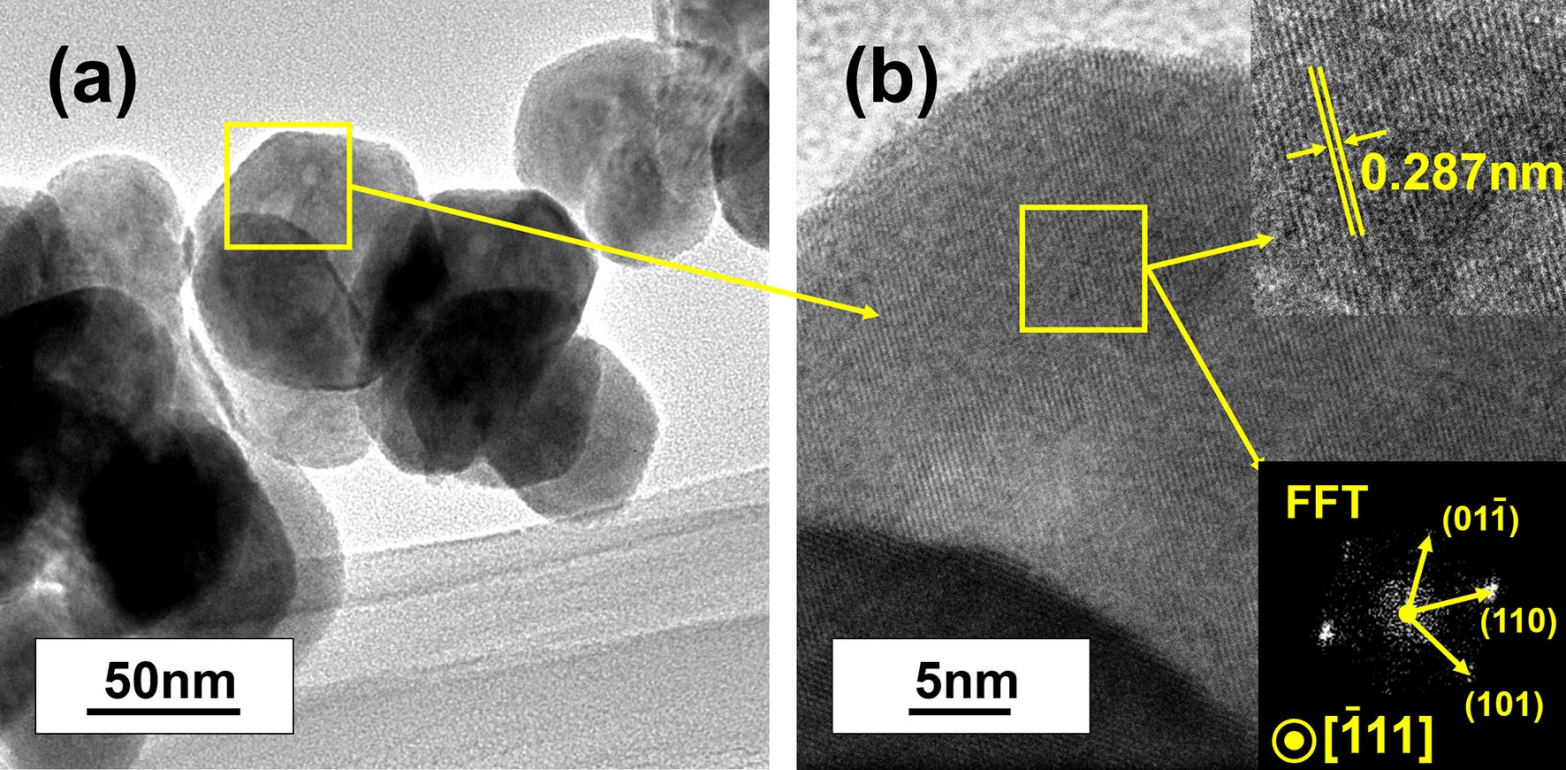
TEM images of BCZT powders synthesized at 180 °C for 60 min: (**a**) TEM image of BCZT powders; (**b**) high-resolution stripes.

Figure 4 shows the TEM images of BCZT powders obtained by MSGH at 180 °C for 60 min. It is clear from Fig. 4a that the boundaries of the particulates are clear and the powder size from TEM image is around 90 nm, which is basically consistent with SEM. Moreover, the powder size obtained here is slightly smaller compared with our previous work (around 105 nm by sol–gel method and 93 nm by sol–gel-hydrothermal method^15,21^) and other literature (around 100 nm by hydrothermal method^22^). Furthermore, from Fig. 4b, fringe features of crystal lattice with a fringe spacing of 0.287 nm corresponding to (110) planes can be observed clearly, proving the good crystallinity of the powders synthesized at 180 °C for 60 min^7^.

Compared with our previous work about low temperature synthetizing BCZT powders by sol–gel-hydrothermal method, MSGH can shorten the reaction time greatly (about 11 h shorter)^15^, which can be attributed to the introduction of microwave in enhancing the reaction efficiency^16^. In addition, compared with other conventional methods, MSGH has a shorter reaction time than solid-state reaction (> 2 h), sol–gel method (> 2 h) and hydrothermal method (> 10 h)^2,7,8^, indicating the advantages of MSGH in rapid synthesis of powders.

**Figure 5:**
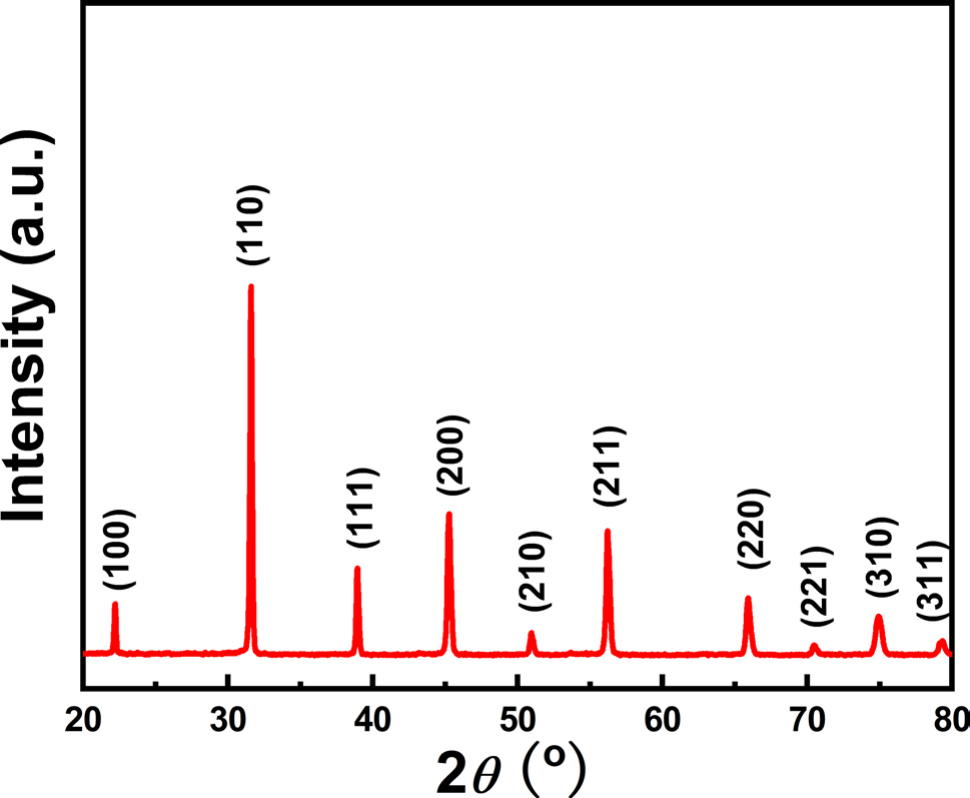
XRD patterns of MSGH derived BCZT ceramic sintered at 1400 °C.

BCZT powders synthesized at 180 °C for 60 min were further sintered at 1400 °C to prepare BCZT ceramics. Figure 5 shows the XRD patterns of BCZT ceramic. All the diffraction peaks show a typical perovskite structure with no impurities, suggesting the high purity of the BCZT ceramic^23,24^. Besides, the sharp diffraction peaks of the XRD pattern indicate great crystallinity of BCZT ceramic sintered at 1400 °C.

**Figure 6:**
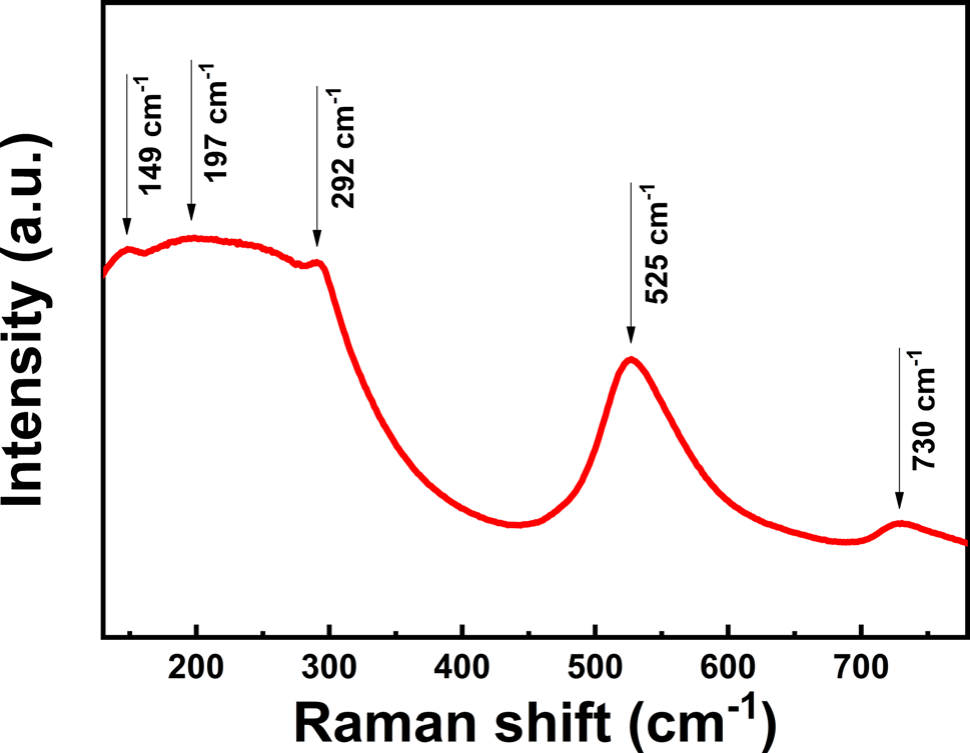
Raman spectra of MSGH derived BCZT ceramic sintered at 1400 °C.

In order to confirm the phase structures of the obtained BCZT ceramics, Raman spectra are measured ranging from 125 cm^−1^ to 775 cm^−1^, which is shown in Fig. 6. Modes at 149, 197, 292, 525, 730 cm^−1^ can be clearly observed respectively. Modes at about 149 and 197 cm^−1^ reflect the existence of the rhombohedral phase^25^, while modes at about 292, 525 and 730 cm^−1^ prove the existence of the tetragonal phase^26^, which agrees well with previous reports^27,28^. Thus, the result of Raman spectra indicates he coexistence of two phases in MPB structure of BCZT ceramics.

**Figure 7:**
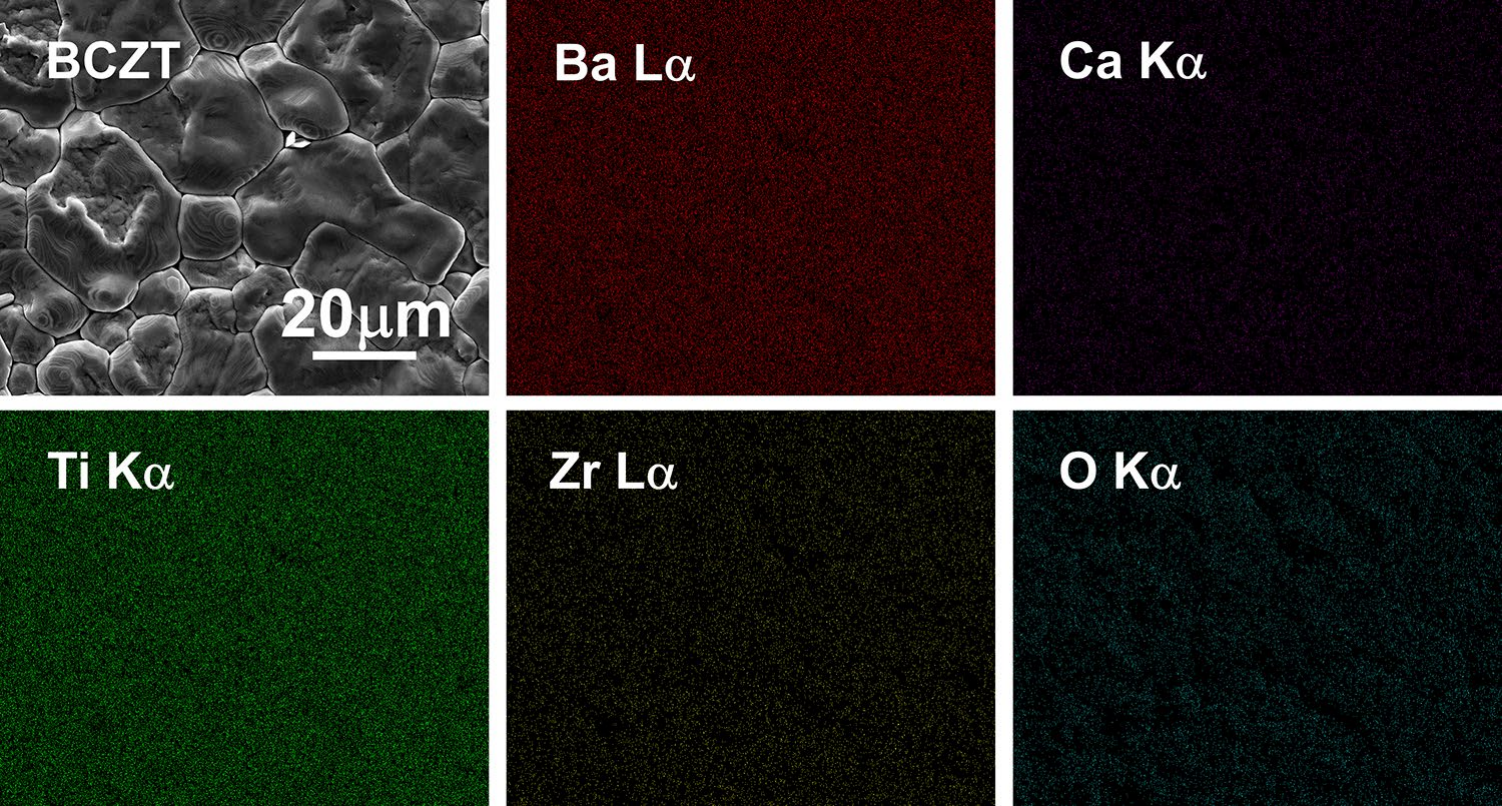
SEM image and EDS spectra of MSGH-derived BCZT ceramic sintered at 1400 °C.

Figure 7 shows the SEM image and EDS spectra of BCZT ceramics. The SEM image shows a homogenous distribution of grains with a dense microstructure and the grain size is measured to be 20–30 μm. The density of the obtained BCZT ceramics is 5.57 g/cm^3^, which is better than other related reports^29,30^. As shown in the EDS spectrums, all elements belonging to BCZT ceramics are uniformly distributed throughout the observed area, without any significant element enrichment areas. Compared with solid-state reaction and sol-gel derived BCZT ceramics, MSGH derived BCZT ceramics have a lower sintering temperature (1400 °C) than those in the related reports (1450–1600 °C)^7,31–33^, which may be attributed to the high activity of BCZT powders prepared by MSGH^34,35^.

**Figure 8:**
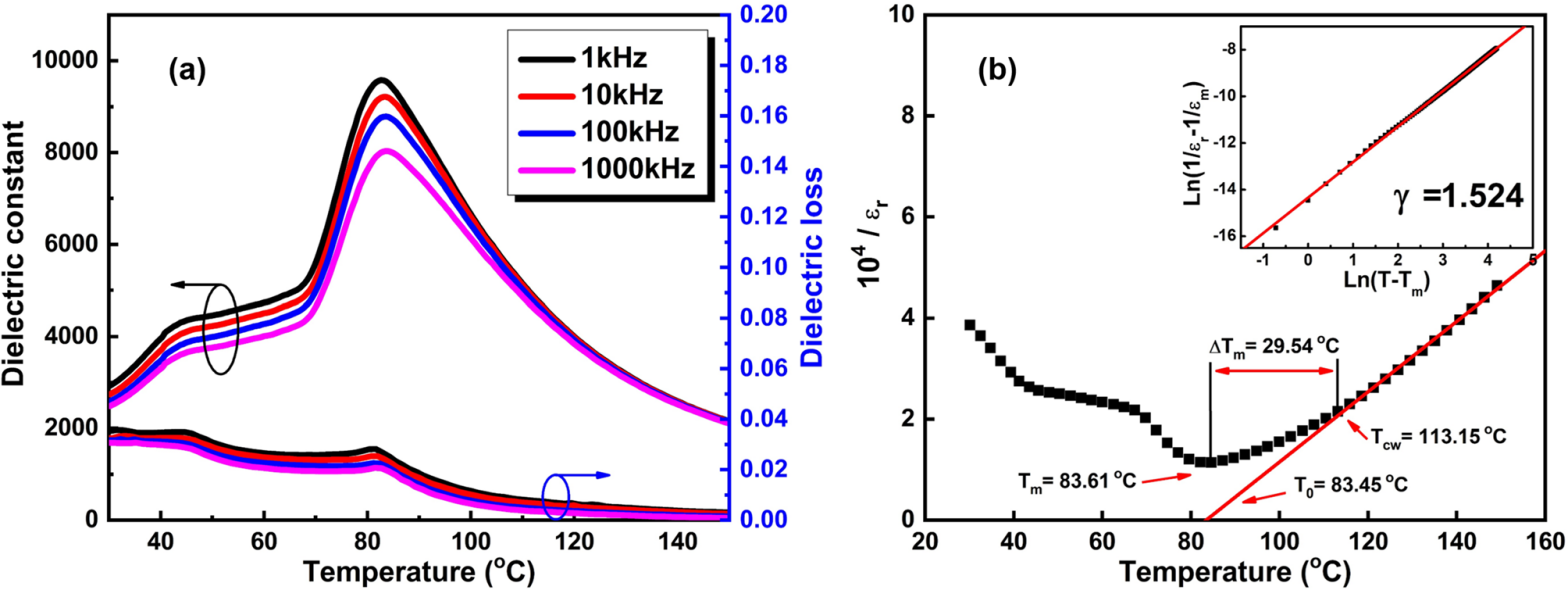
(**a**) Temperature-dependence of dielectric constant and dielectric loss for MSGH-derived BCZT ceramic sintered at 1400 °C, and (**b**) Curie–Weiss fitting curves of MSGH-derived BCZT ceramic sintered at 1400 °C [The inset is plot of ln(T − T_m_) vs. ln(1/ε_r_ − 1/ε_m_)].

Figure 8a shows the temperature vs. dielectric constant (ε_r_) for BCZT ceramics measured at 1 kHz, 10 kHz, 100 kHz and 1000 kHz, respectively. The T_C_ and ε_m_ is measured to be 83.61 °C and 9579 respectively under 1 kHz frequency, and the ε_m_ obtained here is slightly higher than that in other related reports^8,36^. However, an interesting phenomenon can be observed that T_C_ measured in this work is lower than that in other literature^3,7^, which may be attributed to the larger grain size ^37^. Duo to the ferroelectric transition of BCZT ceramics, two dielectric anomalies can be observed, which is related to the phase transition of rhombohedral-tetragonal and tetragonal-cubic^2^. Moreover, a relaxor ferroelectrics phenomenon of strong frequency dispersion and diffuse phase transition could be observed clearly, which is manifested as that not only ε_r_ decreases but also T_C_ moves to higher temperatures area with the increasing frequency^31^. Furthermore, the temperature and frequency—dependence of dielectric loss (tanδ) of the BCZT ceramic is also shown in the Fig. 8a. The relatively low tanδ observed in this work may be ascribed to the less cavities in the dense BCZT ceramic and the lower electron diffusion in the grain boundaries^38,39^.

It is known that the dielectric constant of a normal ferroelectric above the Curie temperature follows the Curie–Weiss law which can be described by:

1 $$1/{\varepsilon} _{{\text{r}}} = (T - T_{0} )/C$$

where T_0_ is the Curie–Weiss temperature and C is the Curie–Weiss constant.

Figure 8b shows the plots of temperature vs. dielectric constant (10^4^/ε_r_ at 100 kHz) fitted to the Curie–Weiss law. For BCZT ceramics obtained through MSGH, T_0_ = 83.45 °C and C = 1.33 × 10^5^ °C were obtained. The C value obtained here is close to the existing literature on BaTiO_3_ (approximately 10^5^ °C), indicates a displacive type phase transition in BCZT ceramics^32^. Moreover, a deviation of dielectric constant from the Curie–Weiss law starting at T_C_ can be seen. The parameter ΔT_m_ which is often used to characterized the degree of the deviation from the Curie–Weiss law and a relaxor-like behavior can be defined as^32,40^:

2 $${T}_{\mathrm{m}}={T}_{\mathrm{cw}}-{T}_{\mathrm{m}}$$

where T_CW_ denotes the temperature at which the dielectric constant starts to deviate from the Curie–Weiss law and T_m_ represents the temperature at which dielectric constant reaches its maximum. A narrower dielectric peak of BCZT ceramic which indicates a weaker diffuse phase transition behavior can be clearly observed as the value of ΔT_m_ (29.54 °C) is lower than that in other literature^41^.

To further describe dielectric behavior of BCZT ceramic, a modified empirical expression proposed by Uchino and Nomura can be given as^42^:

3 $$1/{\varepsilon}_{\mathrm{r}}-1/{\varepsilon}_{\mathrm{m}}={\left(T-{T}_{\mathrm{m}}\right)}/C$$

where C is the Curie–Weiss constant, and γ is a constant implying the degree of diffuse phase transition (1 < γ < 2). A normal Curie–Weiss ferroelectrics can be observed at γ = 1 and an ideal relaxor ferroelectric can be observed at γ = 2. The inset in Fig. 8b shows the plot of ln (T − T_m_) vs. ln (1/ε_r_—1/ε_m_). Depending on the slope of the fitting curve, the value of γ is fitted to be 1.524, reflecting a characteristic of relaxor ferroelectric to some degree.

**Figure 9:**
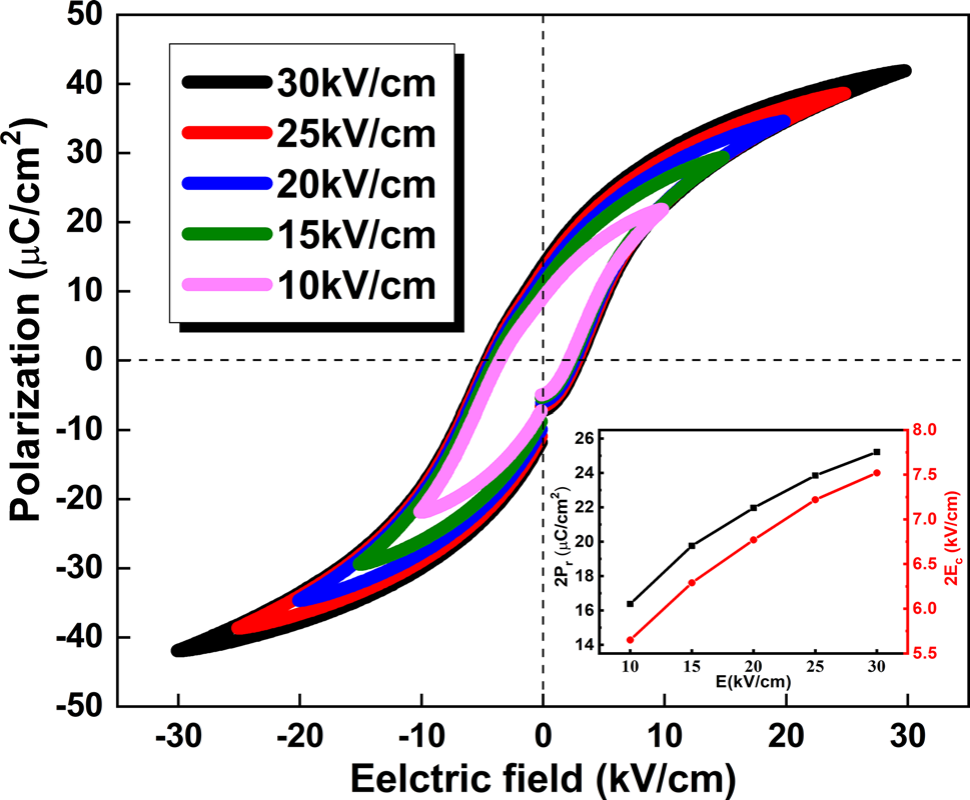
P-E hysteresis loops of MSGH-derived BCZT ceramic sintered at 1400 °C [The inset is remnant polarization (2P_r_) and coercive field (2E_c_) as a function of electric field].

The P-E hysteresis loops of the BCZT ceramics measured under different electric fields are shown in Fig. 9. The inset shows electric fields vs. remnant polarization (2P_r_) and coercive field (2E_c_). With the increasing electrical fields, 2P_r_ as well as 2E_c_ increases gradually and the hysteresis loops tend to be saturated. Well saturated loops with large 2P_r_ (25.22 µC/cm^2^) and moderate 2E_c_ (7.52 kV/cm) were obtained under 30 kV/cm electric field.

Immersed in a silicone oil bath, the BCZT samples coated with silver electrodes were subjected to electrical poling under a certain poling condition (poling field = 35 kV/cm, poling temperature = 300 K, poling time = 30 min). After poling process, the piezoelectric coefficient (d_33_) of the BCZT ceramics are measured, giving the value of 496 pC/N. The large piezoelectric property may be related to the MPB structure in BCZT ceramic and the release of the stress in the domains wall. This may promote the lateral movement of domain walls, even the reorientation and the growth of domains during poling process^43^.

Compare with other reports, MSGH derived BCZT ceramics with better electrical properties (4.66 ~ 23.38 µC/cm^2^^7,44^, 164 ~ 424 pC/N^5,45,46^) can be obtained at a relatively lower sintering temperature (above 1450 °C^5,7,47^), which may attribute to the high activity of BCZT powders prepared by MSGH in this work^22^.

## Conclusion

In summary, Ba_0.85_Ca_0.15_Zr_0.1_Ti_0.9_O_3_ (BCZT) powders were synthesized rapidly by a novel microwave-assisted sol–gel-hydrothermal method (MSGH). The reaction time was shortened to 60 min even at a lower synthesis temperature of 180 °C, as compared with the sol–gel process or sol–gel-hydrothermal methods. BCZT powders were well-crystallized and compositional uniform with fine grains (~ 85 nm). The BCZT ceramic derived from the MSGH-synthesized powders had a dense structure (density 5.57 g/cm^3^) as well as excellent electrical properties (ε_m_ = 9579, d_33_ = 496 pC/N, 2P_r_ = 25.22 µC/cm^2^, E_c_ = 7.52 kV/cm), which was attributed to the high activity of the powders rapidly synthesized by MSGH.
